# Thermo- and Photoresponsive Behaviors of Dual-Stimuli-Responsive Organogels Consisting of Homopolymers of Coumarin-Containing Methacrylate

**DOI:** 10.3390/polym13030329

**Published:** 2021-01-21

**Authors:** Seidai Okada, Eriko Sato

**Affiliations:** Department of Applied Chemistry and Bioengineering, Graduate School of Engineering, Osaka City University, 3-3-138 Sugimoto, Sumiyoshi-ku, Osaka 558-8585, Japan; sed.p.ba.jm@outlook.jp

**Keywords:** organogel, stimuli-responsive gel, lower critical solution temperature, photodimerization

## Abstract

Coumarin-containing vinyl homopolymers, such as poly(7-methacryloyloxycoumarin) (P1a) and poly(7-(2′-methacryloyloxyethoxy)coumarin) (P1b), show a lower critical solution temperature (LCST) in chloroform, which can be controlled by the [2 + 2] photochemical cycloaddition of the coumarin moiety, and they are recognized as monofunctional dual-stimuli-responsive polymers. A single functional group of monofunctional dual-stimuli-responsive polymers responds to dual stimuli and can be introduced more uniformly and densely than those of dual-functional dual-stimuli-responsive polymers. In this study, considering a wide range of applications, organogels consisting of P1a and P1b, i.e., P1a-gel and P1b-gel, respectively, were synthesized, and their thermo- and photoresponsive behaviors in chloroform were investigated in detail. P1a-gel and P1b-gel in a swollen state (transparent) exhibited phase separation (turbid) through a temperature jump and reached a shrunken state (transparent), i.e., an equilibrium state, over time. Moreover, the equilibrium degree of swelling decreased non-linearly with increasing temperature. Furthermore, different thermoresponsive sites were photopatterned on the organogel through the photodimerization of the coumarin unit. The organogels consisting of homopolymers of coumarin-containing methacrylate exhibited unique thermo- and photoresponsivities and behaved as monofunctional dual-stimuli-responsive organogels.

## 1. Introduction

Multi-stimuli-responsive gels have attracted particular attention because of their potential for application in highly functionalized materials [1,2,3]. In most cases, multi-stimuli-responsiveness has been achieved by combining multiple functional groups that respond to a single stimulus [3]. For example, copolymer hydrogels of *N*-isopropylacrylamide (NIPAM) as a temperature-responsive unit and methacrylic acid as a pH-responsive unit are known to be dual-stimuli-responsive gels that respond to temperature and pH [4]. The copolymer hydrogels of NIPAM and acrylic acid can be transformed into PNIPAM-based hydrogels containing pendant crown ether groups by the side-chain reaction of acrylic acid units, and the resulting hydrogels have been applied to lead-sensing materials whose volumes change depending on the Pb^2+^ concentration [5]. In addition to copolymer hydrogels, a nanocomposite hydrogel that achieved thermoresponsiveness and near-infrared-light-responsiveness by combining PNIPAM hydrogel and graphene oxide [6] and hybrid nanoparticles that achieved thermo- and magnetic-field-responsiveness by combining PNIPAM and iron oxide [7] have been reported. Homopolymer hydrogels, consisting of a poly(ferrocenylsilane) backbone with ionic liquid moieties in the side chain, have been reported to be dual-stimuli-responsive; that is, the ionic liquid moiety and the ferrocene moiety are temperature- and redox-responsive, respectively [8,9]. However, only a few studies have reported that multi-stimuli-responsiveness is achieved by a single unit responding to multiple stimuli [10,11]. For example, poly(N, N-dimethylaminoethyl methacrylate) gel is a dual-stimuli-responsive hydrogel in which an N, N-dimethyl aminoethoxycarbonyl moiety responds to temperature and pH; meanwhile, the gel-coated mesh can be used to separate oil and water [11]. 

**Scheme 1:**
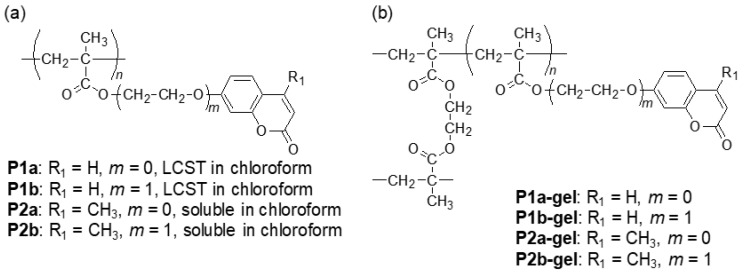
Structures of (**a**) coumarin-containing polymethacrylates and (**b**) gels consisting of coumarin-containing polymethacrylate.

**Scheme 2:**
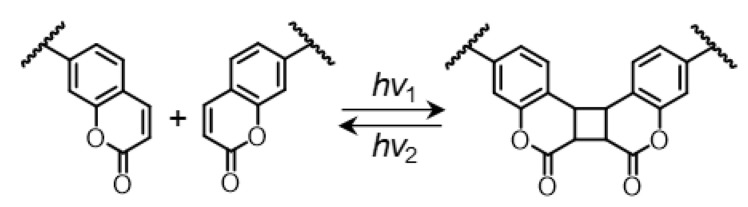
Photochemical [2 + 2] cycloaddition of the coumarin-derivative units.

We have reported that coumarin-derivative homopolymers, such as poly(7-methacryloyloxycoumarin) (**P1a**) and poly(7-(2′-methacryloyloxyethoxy)coumarin) (**P1b**), containing a coumarin moiety in the side chain, exhibit a lower critical solution temperature (LCST)-type phase transition in some halogenated organic solvents with a highly polarized C–H bond, such as chloroform (Scheme 1a) [12]. The LCST of the coumarin derivative polymers is affected by the changes in the contribution of the intermolecular interactions, such as the CH/π and π/π interactions between solvent polarized C-H bonds and polymers with a π-conjugated side group, depending on the temperature [12]. The phase-transition temperature of the polymer solution can be controlled through photoirradiation [12], owing to the progress of the [2 + 2] cycloaddition of the coumarin-derivative units (Scheme 2) [13]. Therefore, it can be stated that coumarin-derivative polymers act as monofunctional dual-stimuli-responsive polymers in which one substituent (coumarin unit) responds to temperature and light. When a single functional group responds to multiple stimuli, stimulus-responsive sites can be introduced in polymers with high density and uniformity. Thus, a highly sensitive and uniform response is expected compared with that of a multi-stimulus-responsive material composed of various functional groups. The phase-transition temperature of the coumarin-derivative polymers can also be controlled over a wide range through various methods, such as, copolymerization with solvophobic or solvophilic comonomers [14] or the addition of a low-molecular-weight aromatic compound, which interacts with the coumarin moiety of the polymer side-chain [15]. Therefore, various applications of functional materials have been performed over wide temperature ranges, such as the thermoresponsive stationary phase for chromatography, which uses chloroform as a mobile phase and a flow controller as a microchannel for water-inhibited reactions. Considering these applications, it is essential to clarify the stimulus-responsive behavior of the coumarin-derivative polymer-based organogels, in addition to the solutions. Although the stimuli-responsive behavior of hydrogels and their derivatives, i.e., gels swollen by the mixed solvents of water and water-miscible organic solvents, have been well investigated from scientific [16,17,18] and technological [19,20,21,22,23,24] perspectives, there is a lack of information on organogels swollen by non-protic organic solvents. In this study, we investigated the thermo- and photoresponsive behaviors of coumarin-containing polymer-based organogels (**P1a-gel** and **P1b-gel**) and clarified their performance as monofunctional dual-stimuli-responsive gels (Scheme 1b).

## 2. Materials and Methods

### 2.1. Materials

Coumarin-containing methacrylates, i.e., 7-methacryloyloxycoumarin (**1a**), 7-(2′-methacryloyloxyethoxy)coumarin (**1b**), 7-methacryloyloxy-4-methylcoumarin (**2a**), and 7-(2′-methacryloyloxyethoxy)-4-methylcoumarin (**2b**), were synthesized according to the process described previously [25]. In this study, 2,2′-azobis(isobutyronitrile) (AIBN, Wako Pure Chemicals Co., Ltd., Osaka, Japan) was recrystallized from methanol. Chloroform (>99.7%, except for ethanol) containing 0.5–0.9% ethanol, (Wako Pure Chemicals Co., Ltd., Osaka, Japan) was used as received. Ethylene glycol dimethacrylate (EGDMA, >97%, Wako Pure Chemicals Co., Ltd. Osaka, Japan) and cyclohexyl methacrylate (>97%, Wako Pure Chemicals Co., Ltd. Osaka, Japan) were distilled under reduced pressure. All other reagents and solvents were used without further purification or were purified according to conventional methods.

### 2.2. Synthesis of Coumarin-Containing Organogels

All polymerizations were performed through the free-radical polymerization of coumarin-containing methacrylates, **1a**, **1b**, **2a**, or **2b**, in the presence of EGDMA as a cross-linker (Scheme 1b). As a representative method, the synthesis of **P1b**-**gel** is described below. In a Pyrex glass tube (diameter = 15 mm) with a flat bottom, **1b** (274 mg, 1.0 × 10^−3^ mol), EGDMA (1.98 mg, 1.0 × 10^−5^ mol), and AIBN (0.82 mg, 5.0 × 10^−6^ mol) in 1,1,2,2-tetrachloroethane (1 mL) were added using the stock solutions of EGDMA (1.0 × 10^−4^ mol/L) and AIBN (5.0 × 10^−5^ mol/L). The solution was degassed by bubbling Ar gas for 30 min, and the tube was sealed under an Ar atmosphere. Polymerization was performed at 60 °C for 24 h. The height of the gel was approximately 5 mm immediately after polymerization. To remove unreacted reagents and exchange 1,1,2,2-tetrachloroethane with chloroform, the synthesized gel was immersed in chloroform for 12 h at room temperature (22 °C) by changing the chloroform every 3 h. All gels were transparent both before and after the substitution of 1,1,2,2-tetrachloroethane to chloroform. **P1b-gel**, used for photopatterning, was similarly prepared at the bottom of the vial to a thickness of approximately 1 mm after polymerization. As a reference gel, poly(cyclohexyl methacrylate)-gel was similarly synthesized. In all cases, the gel fraction was almost 100%. The IR spectra of **P1a-gel** and **P1b-gel** were measured, and the characteristic peaks of a coumarin moiety at 1400 cm^−1^ (*cis* C=C stretching), 1512 cm^−1^ (ring C=C stretching), 1610 cm^−1^ (ring C=C stretching), and 1725 cm^−1^ (C=O stretching) were observed [26]. It is reasonable to expect that the structure of the gels shown in Scheme 1b was formed, taking into account that free-radical polymerization of the coumarin-containing methacrylates, in the absence of EGDMA, resulted in the formation of the corresponding polymethacrylates (the average molecular weights and molecular weight distributions of the linear polymers were tens of thousands and 1.2–2.1, respectively) (Figure S1a) [12]. To obtain information on the mechanical strength of the gels, a **P2a-gel** swollen by chloroform was compressed, and the failure occurred at over 80% compression. The stress-strain profile typically observed for conventional gels was obtained (Figure S1b). 

### 2.3. Observation of Organogels during Heating

A hot plate (HP-19U300, Koike Precision Instruments, Itami, Japan) was used to observe the shape of the organogels during the heating process. The gel was immersed in chloroform in a Petri dish and placed on a hot plate, under which a 5 mm square of graph paper was laid as a size guide. **P1a-gel** and **P1b-gel** swollen at −30 °C were used. The temperature of the hot plate was increased from room temperature to 50 °C. The temperature of chloroform was measured every minute using a mercury thermometer, and the heating rate was estimated to be approximately 3 °C/min. The top-view images of the gels were recorded using a digital camera. 

### 2.4. Determination of Gel Fraction and Degree of Swelling

The gel fraction and degree of swelling of the organogels were calculated using Equations (1) and (2), respectively. The symbols *W*_1_, *W*_2_, and *W*_3_ represent the weights of the monomer, insoluble fraction, and swollen gel at equilibrium at a predetermined temperature, respectively. To determine *W*_3_ for the equilibrium degree of swelling, the gel was allowed to stand in chloroform for 100 h or longer, and the weight change after an additional 24 h was confirmed to be ± 5% or less. Parameters *ρ*_1_ and *ρ*_2_ represent the densities of chloroform (1.48 g/cm^3^) and polymer (assumed to be 1.0 g/cm^3^ to simplify the calculation), respectively.

(1) $$Gel\;fraction\;\left(wt\%\right)=W_{2}\;/W_{1}\;\times 100$$

(2) $$Degree\;of\;swelling\;\left(wt\%\right)=\frac{\left(W_{3}-W_{2}\right)\rho_{2}}{W_{2}\rho_{1}}\times 100\%$$

### 2.5. Photopatterning

The photoreactions were performed under atmospheric conditions via photoirradiation using a high-pressure Hg lamp (MSU-6, 250 W, Moritex, Asaka, Japan) with a strong emission at 313 nm. A heat cut filter (UVF-350) to eliminate light between 400 and 800 nm and a Pyrex glass filter (2 mm thickness) to eliminate light below 290 nm were used. The spectral distribution of the light source is shown in Figure S2. The UV–Vis absorption spectra of **P1a** in chloroform are shown in Figure S3 as representative data showing the absorption region of the coumarin moiety, which undergoes [2 + 2] cycloaddition (Scheme 2). The coumarin-containing organogels in Pyrex glass vials with a flat bottom were placed 10 cm from the light source, and a light intensity of approximately 15.4 mW/cm^2^ was measured using an accumulated UV meter (UIT-250, Ushio Inc., Tokyo, Japan) equipped with a UVD-S365 optical receiver (330–390 nm). Aluminum foil was used as the photo mask. Photoirradiation was performed twice for 5 min each by changing the masked area (Figure S4).

## 3. Results and Discussion

### 3.1. Thermoresponsive Behavior of Coumarin-Containing Organogels

**Figure 1:**
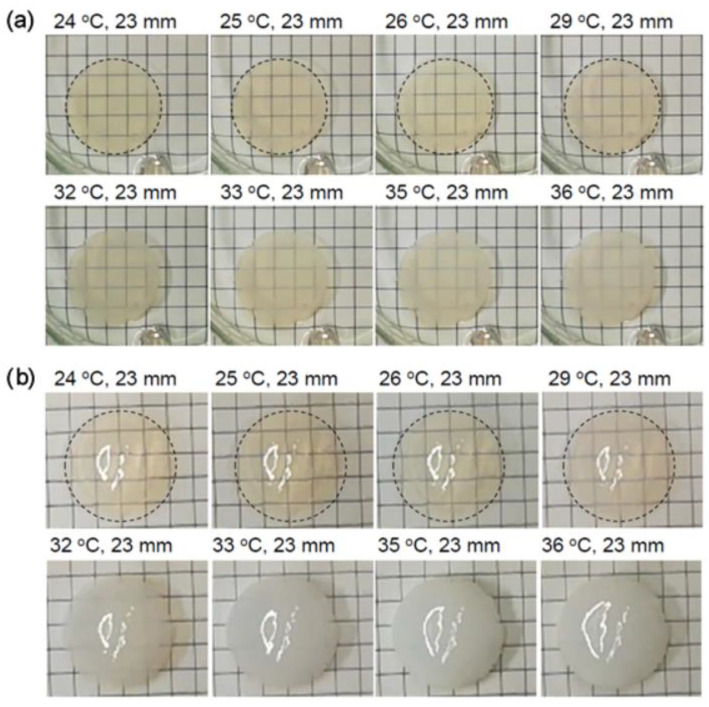
Top-view photographs of (**a**) P1a-gel and (**b**) P1b-gel with a pellet shape in chloroform during the heating process (~3 °C/min). The values inserted above each picture denote the temperature and the diameter of the gels. Dashed circles were drawn as visual guides for the outer shape of the gels. Each square of the grid corresponds to 5 mm.

To obtain an understanding of the thermoresponsive behavior of coumarin-containing gels, the shape changes of the **P1a-gel** and **P1b-gel** consisting of the polymers that exhibit LCST in chloroform were investigated during a relatively rapid heating process (approximately 3 °C/min). Similar experiments were conducted using **P2a-gel** and **P2b-gel**, consisting of the polymers that did not exhibit LCST (soluble up to the boiling point) in chloroform due to the steric hindrance of the methyl group at the 4-position of the coumarin ring. The results of the experiments were then compared. Informative pictures taken during the heating process of **P1a-gel** and **P1b-gel** were selected and are illustrated in Figure 1. Pictures were recorded each minute and are shown in Figures S5 and S6. In both cases, turbidity was observed at approximately 30 °C with increasing temperature. This turbidity is expected to be due to the aggregation of the polymer chains by LCST. During this relatively rapid heating process at approximately 3 °C/min, significant volume shrinkage, which is typically observed for thermoresponsive hydrogels such as PNIPAM gel [16,17], was not observed for either **P1a-gel** or **P1b-gel**. In the case of **P2a-gel** and **P2b-gel**, neither the transparency nor the diameter of the gels changed during the heating process up to 50 °C (Figure S7). The corresponding linear polymers to **P2a-gel** and **P2b-gel**, i.e., **P2a** and **P2b**, were soluble in chloroform up to the boiling point [12], and aggregation of the polymer chains did not occur under the current experimental conditions. 

### 3.2. Deswelling Behavior during the Isothermal Process

**Figure 2:**
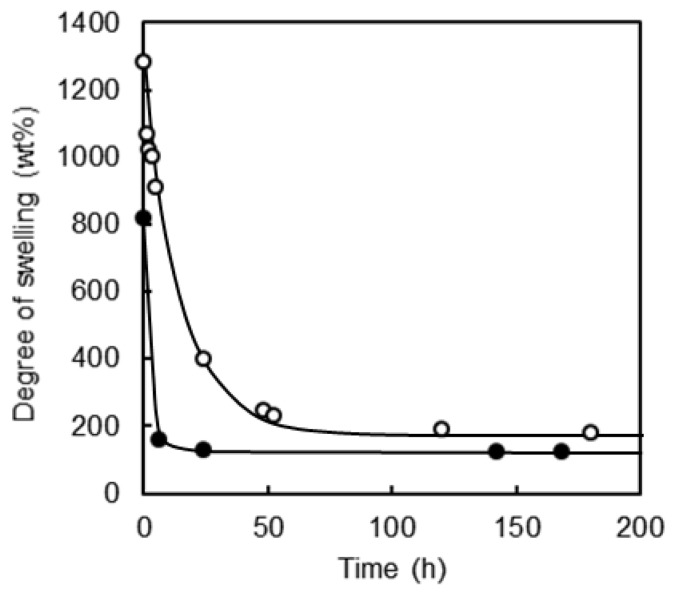
Deswelling behaviors of **P1a-gel** (solid circle) and **P1b-gel** (open circle) after soaking in chloroform at 42 °C.

Figure 2 shows the time change of the degree of swelling of **P1a-gel** and **P1b-gel** at 42 °C, which is approximately 10 °C higher than the temperature at which they become turbid during the heating process. The gels that experienced sufficient swelling at −30 °C were used. The degree of swelling of **P1a-gel** and **P1b-gel** decreased remarkably with increasing immersion time at 42 °C, reaching a shrunken state in approximately 24 h for **P1a-gel** and approximately 100 h for **P1b-gel** (Figure 2). It was reported that in the case of PNIPAM hydrogels with a diameter of 15 mm, several weeks were required to reach the shrinkage equilibrium [27,28]. This is because the internal solvent release is inhibited by the skin layer formed on the gel surface by the aggregation of the polymer chains at a temperature higher than the LCST. The gel used for our current experiment was synthesized with a diameter of 15 mm and thickness of 5 mm and required a long time to reach shrinkage equilibrium due to the formation of a skin layer, as in the case of the PNIPAM gel. 

**Figure 3:**
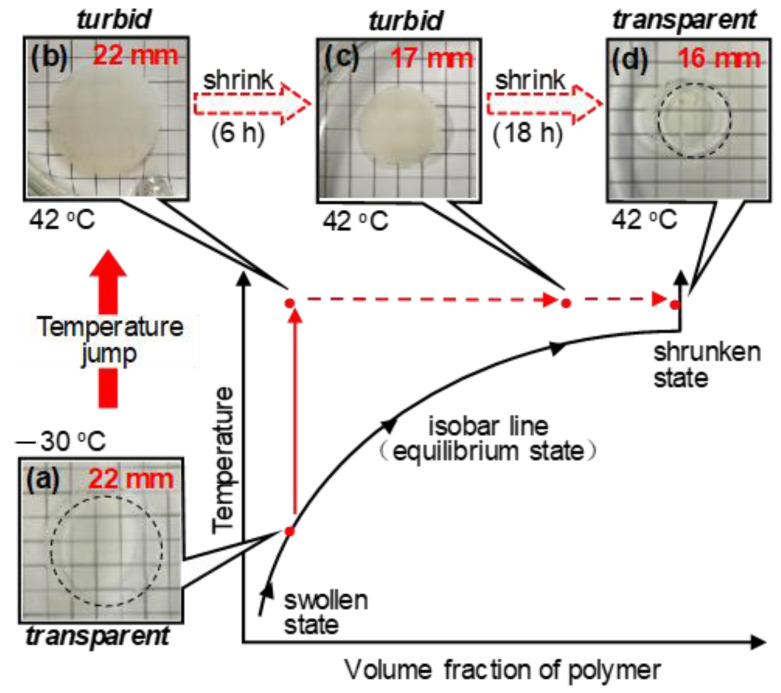
Photographs of **P1a-gel** (**a**) sufficiently swollen at −30 °C and (**b**–**d**) during the isothermal deswelling process at 42 °C: (**b**) immediately after changing to 42 °C, (**c**) after 6 h, and (**d**) after 18 h. The inserted value in the photographs denotes the diameter of the **P1a-gel**. Dashed circles were drawn as visual guides for the outer shape of the gels. Each square of the grid corresponds to 5 mm. The schematic phase diagram (Temperature vs. Volume fraction of polymer) was reported for the thermoresponsive hydrogels by Shibayama et al. [18].

**Figure 4:**
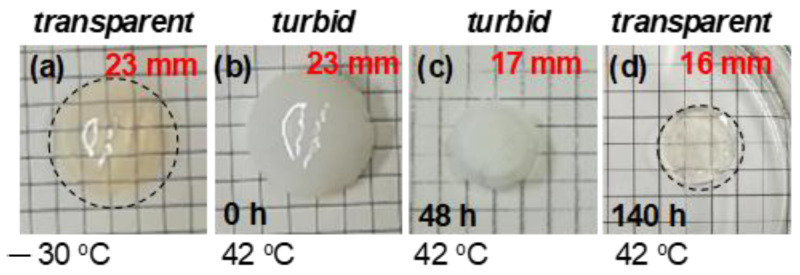
Photographs of P1b-gel (**a**) sufficiently swollen at −30 °C and (**b**–**d**) during the isothermal deswelling process at 42 °C: (**b**) immediately after changing to 42 °C, (**c**) after 48 h, and (**d**) after 140 h. The inserted value in the photographs denotes the diameter of P1b-gel. Dashed circles were drawn as visual guides for the outer shape of the gels. Each square of the grid corresponds to 5 mm.

The shape changes of the **P1a-gel** during the rapid temperature increase from −30 °C to 42 °C, in which the temperature was maintained at 42 °C until the equilibrium degree of swelling was reached, are shown in Figure 3. The rapid temperature increase, i.e., the temperature jump, resulted in an unstable phase separation at the surface of the gel (Figure 3a,b), in which the surface became turbid. In addition, the decrease in diameter was negligible immediately after immersion. At 42 °C, the gel gradually shrank by releasing chloroform from its interior over a long period (Figure 3c), ultimately reaching a more stable and transparent shrunken state (Figure 3d). Notably, the slow shrinkage of the currently studied relatively large gels is partially due to the formation of a skin layer to prevent solvent release, as mentioned above. Similar transparency and volume changes were also observed for the **P1b-gel** (Figure 4). **P1b-gel** did not reach the shrunken state, even after 48 h at 42 °C, whereas **P1a-gel** did within 18 h. The result that **P1b-gel** took a longer time to reach a shrunken state than **P1a-gel** is in agreement with the observation in Figure 2.

**Figure 5:**
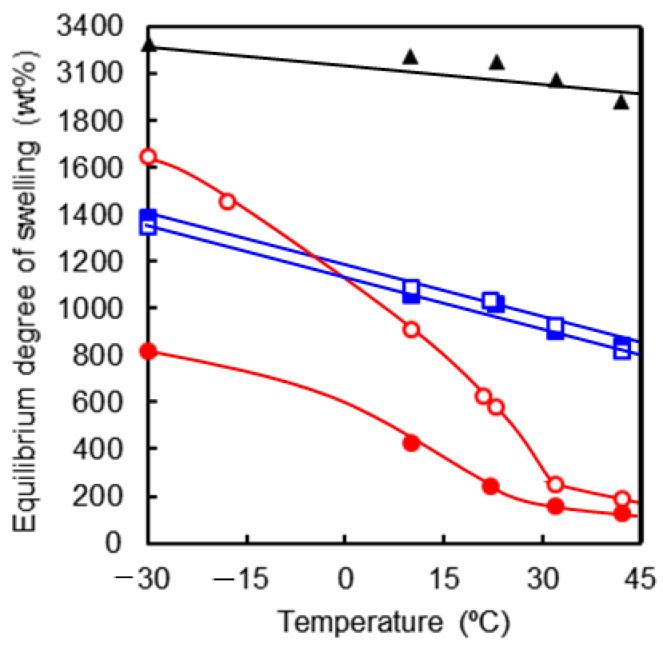
Temperature dependence of the equilibrium degrees of swelling of P1a-gel (red solid circle), P1b-gel (red open circle), P2a-gel (blue solid square), P2b-gel (blue open square), and poly(cyclohexyl methacrylate)-gel (black solid triangle) in chloroform.

Figure 5 shows the temperature dependence of the equilibrium degree of swelling of the coumarin-containing organogels. The equilibrium degree of swelling of **P1a-gel** and **P1b-gel**, the main chain of which consists of the LCST polymer, showed a significant and non-linear decrease with increasing temperature, and the changes in the degree of swelling decreased at temperatures above 30 °C. These observations suggest that **P1a-gel** and **P1b-gel** exhibited LCST and reached a shrunken state at a higher temperature. In contrast, the equilibrium degree of swelling of the **P2a-gel** and **P2b-gel**, the main chain of which consists of a non-LCST polymer, linearly decreased with increasing temperature, and the extent of the decrease was much smaller than those in the cases of **P1a-gel** and **P1b-gel**. A similar decrease in the equilibrium degree of swelling was observed for poly(cyclohexyl methacrylate)-gel, in which the corresponding linear polymer was soluble up to the boiling point of chloroform; this constant decrease is not thought to be derived from the LCST. These results indicate that the coumarin-containing organogels show swelling/deswelling behavior reflecting the thermoresponsiveness of linear polymers and demonstrate a significant temperature dependence of the equilibrium degree of swelling. Moreover, the temperature dependence of the equilibrium degree of swelling of **P1a-gel** and **P1b-gel** drastically changes below and above the cloud point of the corresponding linear polymer solution. The cloud points of **P1a** and **P1b** are described in the next section. This phenomenon can be regarded as a volume phase transition, which is a rare observation for non-aqueous systems, such as poly(*N*-propargyl amide)-based gels swollen by chloroform [29] and poly(benzyl methacrylate)-based gels swollen by ionic liquids [30].

When the equilibrium degrees of swelling of **P1a-gel** and **P1b-gel** were compared below 30 °C, the equilibrium degree of swelling of **P1b-gel** was higher than that of **P1a-gel**. In the case of the linear polymers, the cloud points of the 0.3 wt% chloroform solution of **P1a** and **P1b** were 28 and 37 °C, respectively [12], and **P1b** underwent a phase separation at a higher temperature than **P1a**. In other words, in the case of **P1b**, the interaction between the polymer and the solvent was more dominant than that between the polymer chains, compared with the case of **P1a**. It appears that the equilibrium degree of swelling of **P1a-gel** and **P1b-gel** reflects the interaction between the corresponding linear polymer and the solvent. 

### 3.3. Reversibility of Thermoresponsive Cehavior of Coumarin-Containing Organogels

**Figure 6:**
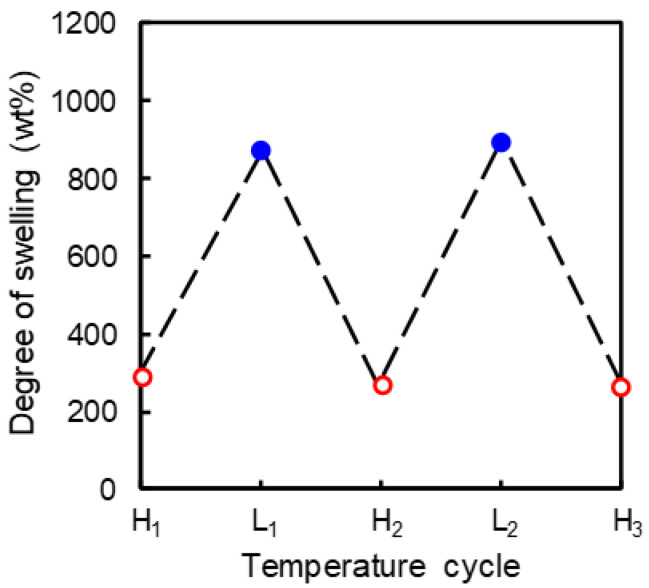
Reversibility of the degree of swelling of P1b-gel after soaking in chloroform at 45 °C for 24 h (Hn, red open circle) and at −30 °C for 24 h (Ln, blue solid circle).

The equilibrium degree of swelling of **P1a-gel** and **P1b-gel** significantly decreased as the temperature increased. To confirm whether the shrinking behavior was reversible, **P1b-gel** was repeatedly immersed in chloroform at 45 °C and −30 °C every 24 h (Figure 6). At 45 °C, **P1b-gel** shrunk, and the degree of swelling was as low as 260–290 wt%. Furthermore, **P1b-gel** was significantly swollen at −30 °C, with a degree of swelling as high as 870–890 wt%. Furthermore, although the examined number of cycles was small and the stability of the gels was not clear, it was determined that **P1b-gel** reversibly shrinks and swells at least three times by increasing and decreasing the temperature, respectively. 

### 3.4. Photopatterning of Thermoresponsive Sites

**Figure 7:**
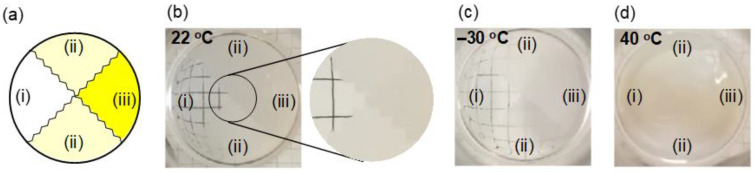
(**a**) Schematic illustration of photopatterning on a gel with different irradiation time ((i) 0 min, (ii) 5 min, and (iii) 10 min) and (**b**–**d**) photographs of the photopatterned P1b-gel at different temperatures: (**b**) at 22 °C with the enlarged view of the boundary area, (**c**) at −30 °C after maintaining for 0.5 h, and (**d**) at 40 °C after maintaining for 0.5 h.

Coumarin-derivative polymers, such as **P1a,** have a photoresponsive coumarin moiety in the side chain of each monomer unit and undergo [2 + 2] cycloaddition, i.e., photodimerization, via UV irradiation (Scheme 2) [13]. We have reported that the phase-transition temperature of the chloroform solution of **P1a** decreases as photodimerization advances, and the thermoresponsive coumarin-containing homopolymers can be regarded as monofunctional dual-stimuli-responsive polymers that respond to temperature and light [12]. Accordingly, it is expected that different thermoresponsive sites can be fabricated on the surface of thermoresponsive coumarin-containing organogels by photopatterning. As a thermoresponsive coumarin-containing organogel, a thin disk-like **P1b-gel** was synthesized and subjected to UV irradiation at room temperature to achieve the photodimerization using a high-pressure Hg lamp with a strong emission at 313 nm. The IR spectra of the surface of **P1b-gel** before and after photoirradiation confirmed that photodimerization had taken place, that is, a new shoulder peak assignable to a nonconjugated C=O group in the cyclobutene ring in the coumarin dimer was observed at 1768 cm^−1^ (Scheme 2 and Figure S8) [26]. Moreover, in our previous work using a linear polymer solution and thin film, the progress of the photodimerization of the coumarin unit under similar conditions was monitored by UV–Vis spectra changes [25]. The concentration of the **1b** unit in the synthesized gel was approximately 20 times higher than the concentration of the coumarin moiety in a linear polymer solution, in which intermolecular dimerization of the coumarin unit took place by photoirradiation [31]. Therefore, it is expected that photodimerization of the coumarin unit in the different primary chain proceeds under the current experimental conditions. The non-irradiated area and two other areas were irradiated for different durations, i.e., 5 and 10 min, and were prepared on the single **P1b-gel** (Figure 7a and Figure S4). At room temperature, the non-irradiated area remained transparent; however, the areas irradiated for 5 and 10 min became turbid (Figure 7b). In the enlarged image of the boundary of the areas subjected to different irradiation durations, a sawtooth structure on the order of millimeters caused by the cut edge of the aluminum foil used for the photo mask can be observed. Although it is not apparent, the boundary between the areas irradiated for 5 and 10 min was also recognizable, whereby the area irradiated for 10 min was more turbid than the area irradiated for 5 min, according to visual observation. The observations in Figure 7b suggest that the phase-transition temperature of the dimerized surface of the **P1b-gel** was lower than room temperature and decreased with increasing irradiation time, i.e., with the progress of dimerization. The photodimerization only proceeded near the surface of the gel that was nearest to the UV light source, and no significant changes were observed in the degree of swelling of the gel before and after photodimerization (Figure S9).

To examine the thermoresponsivity, the photoirradiated **P1b-gel** was cooled to −30 °C for 0.5 h to change the temperature of the entire gel (Figure 7c). The appearances of the sites irradiated for 0 and 10 min were unchanged from those observed at 22 °C, i.e., they were transparent and turbid, respectively. However, the site irradiated for 5 min became partly transparent. Within the site irradiated for 5 min, the area closer to the site irradiated for 10 min remained turbid, and the area closer to the non-irradiated site became transparent. A possible explanation for this unique observation is that the time required to reach the equilibrium state, where gels become transparent, is longer for the region with higher cross-linking density because of less chain mobility; therefore, the site with higher cross-linking density delays the change of the adjacent region with a lower cross-linking density to a transparent state. Another possible explanation is that the scattered UV light penetrated the neighboring area and increased the degree of photodimerization in the area closer to the site irradiated for 10 min. When the cooled **P1b-gel** was heated to 40 °C, all sites became turbid within 0.5 h (Figure 7d). This is because a rapid increase in the temperature results in phase separation, where gels promptly become turbid, as illustrated in Figure 3 and Figure 4. The trends showing a decrease in the clouding temperature with increasing UV irradiation time are summarized in Table S1. These results confirm that the sites responding to different temperatures can be patterned by the application of UV light irradiation to a coumarin-containing organogel with a resolution on the order of millimeters. 

## 4. Conclusions

Coumarin-containing organogels consisting of LCST polymers, i.e., **P1a** and **P1b**, and non-LCST polymers, i.e., **P2a** and **P2b**, were synthesized, and their thermo- and photoresponsive behaviors in chloroform were investigated in detail. Transparent **P1a-gel** and **P1b-gel** sufficiently swollen at −30 °C showed a phase separation (turbid) without shrinking through a temperature jump to 42 °C and reached a shrunken state (transparent) after 24 and 100 h, respectively. The equilibrium degree of swelling of **P1a-gel** and **P1b-gel** decreased non-linearly with increasing temperature and reached a shrunken state at temperatures above 30 °C. The significant volume change of the **P1b-gel** was thermally reversible. In contrast, in the case of **P2a-gel** and **P2b-gel**, transparency was maintained during a relatively rapid heating process, and the equilibrium degree of swelling linearly and slightly decreased with increasing temperature. When the **P1b-gel** was irradiated with UV light to perform photodimerization of the coumarin unit, the temperature at which the gel surface became cloudy decreased with increasing irradiation time, i.e., with the progress of photodimerization. Thus, different thermoresponsive sites were successfully photopatterned on the **P1b-gel**.

## Supplementary Materials

The following are available online at https://www.mdpi.com/2073-4360/13/3/329/s1. Figure S1. (a) ^1^H NMR spectra of **P1a**, **P1b**, **P2a**, and **P2b**, and (b) stress-strain curve of **P2a gel**: compression rate = 5 mm/min; Figure S2. Spectral distribution of the light source; Figure S3. UV-Vis absorption spectrum of **P1a** in chloroform; Figure S4. Schematic diagram of photopatterning: (i) non-irradiated, (ii) 5-min irradiated, and (iii) 10-min irradiated sites; Figure S5. Top-view photographs of **P1a gel** with a pellet shape in chloroform during the heating process (ca. 3 °C/min). The values inserted above each picture denote the temperature Each square of the grid corresponds to 5 mm; Figure S6. Top-view photographs of **P1b gel** with a pellet shape in chloroform during the heating process (ca. 3 °C/min). The values inserted above each picture denote the temperature Each square of the grid corresponds to 5 mm; Figure S7. Top view photographs of (a) **P2a gel**, and (b) **P2b gel** with a pellet shape in chloroform during the heating process (ca. 3 °C/min). The values inserted in each picture denote temperature and the diameter of the gels. Dashed circles were drawn as visual guides for the outer shape of gels. Each square of the grid corresponds to 5 mm. Figure S8. IR spectra of the surface of **P1b-gel** before (a) and after (b) photoirradiation. The arrow (i) shows a new shoulder peak at 1768 cm^−1^ assignable to a nonconjugated C=O group in the cyclobutene ring in the coumarin dimer. Figure S9. Time dependence of the degree of swelling of **P1b-gel** at 42 °C before (●) and after 5-min UV irradiation (○). Table S1. Turbidity characteristics of P1b gel at each temperature.
