# Supplementary material for: Thermo- and Photoresponsive Behaviors of Dual-Stimuli-Responsive Organogels Consisting of Homopolymers of Coumarin-Containing Methacrylate
Source: Polymers (Basel). 2021 Jan 21;13(3):329. doi: 10.3390/polym13030329 (PMC7864332; doi:10.3390/polym13030329)
Supplement: Supplementary file 1 [file polymers-13-00329-s001.pdf]

Supporting Information

Thermo- and Photoresponsive Behaviors of Dual-Stimuli-Responsive Organogels Consisting of Homopolymers of Coumarin-Containing Methacrylate

*Seidai Okada and Eriko Sato**

Department of Applied Chemistry and Bioengineering, Graduate School of Engineering, Osaka City University, 3-3-138 Sugimoto, Sumiyoshi-ku, Osaka 558-8585, Japan

Corresponding author: satoeriko@osaka-cu.ac.jp

(a)

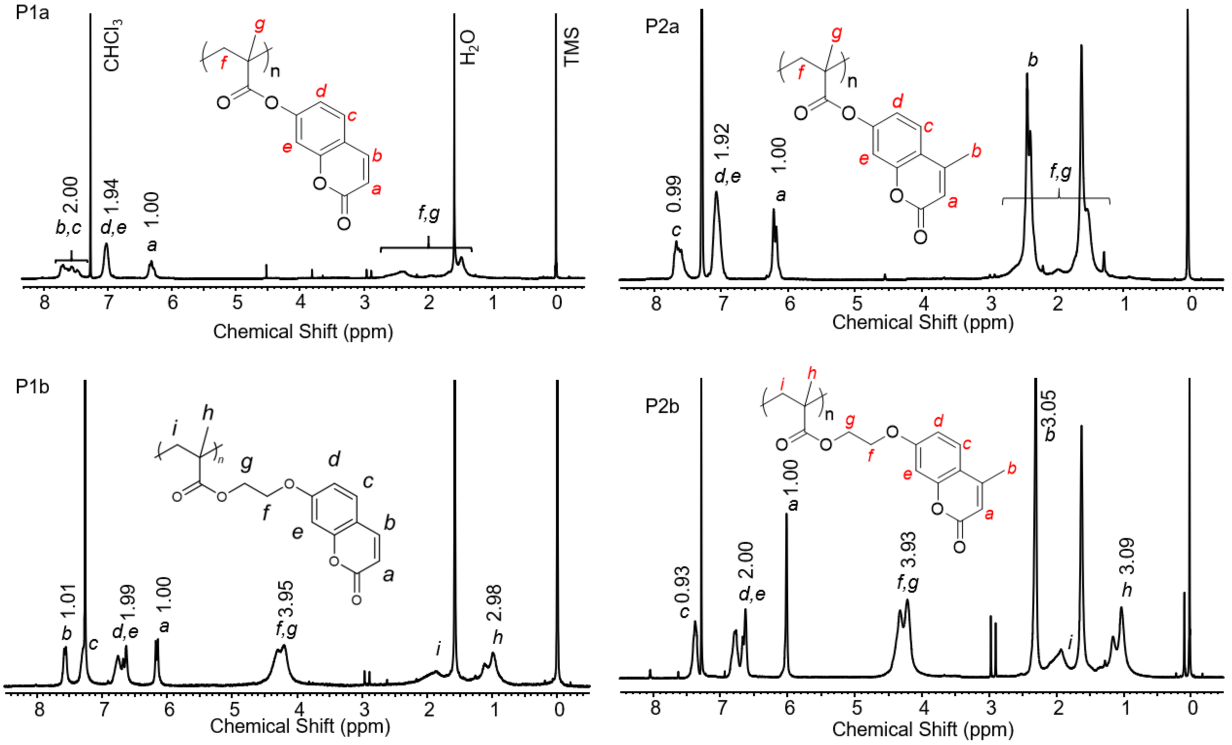

(b)

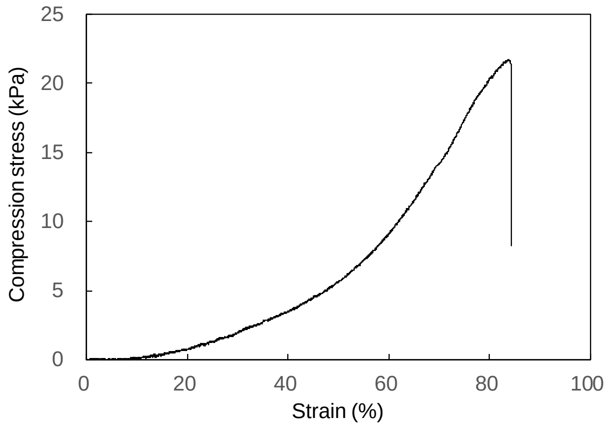

Figure S1. (a) ^{1}H NMR spectra of **P1a**, **P1b**, **P2a**, and **P2b**, and (b) stress-strain curve of **P2a gel**: compression rate = 5 mm/min.

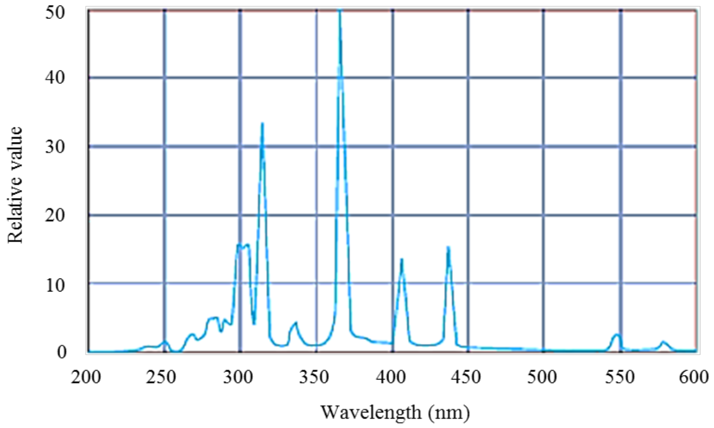

Figure S2. Spectral distribution of the light source.

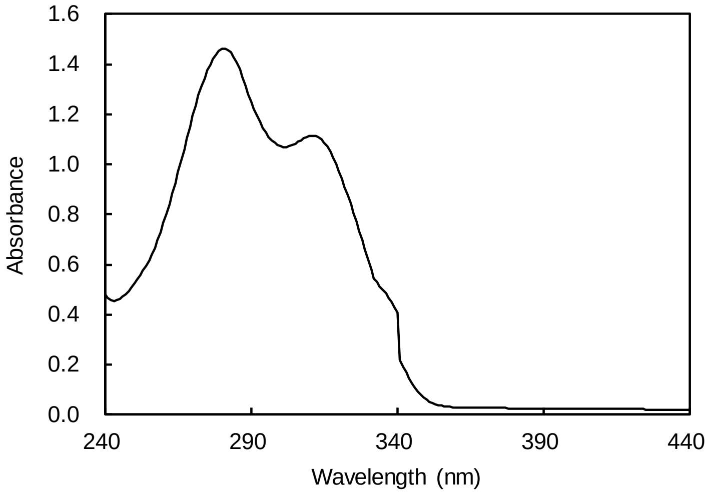

Figure S3. UV-Vis absorption spectrum of **P1a** in chloroform.

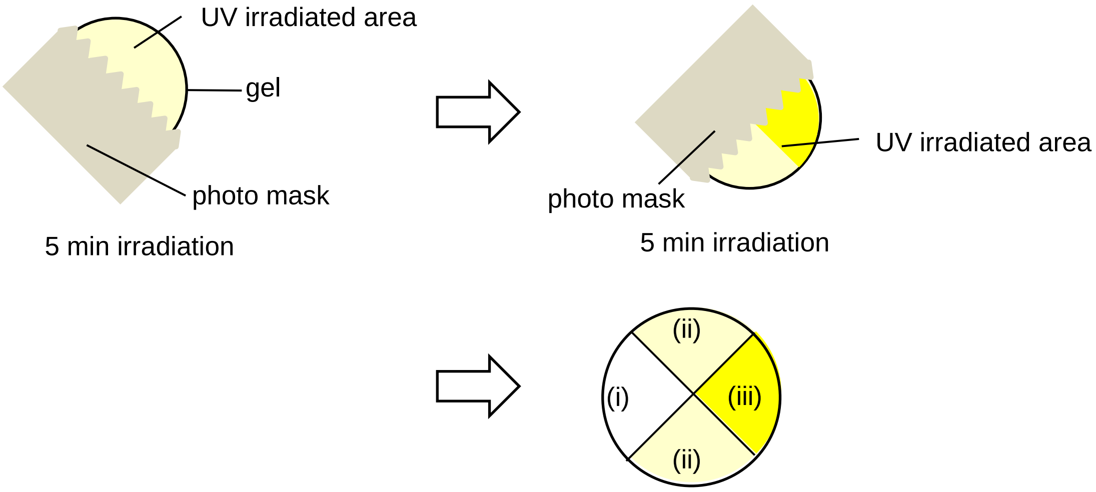

Figure S4. Schematic diagram of photopatterning: (i) non-irradiated, (ii) 5-min irradiated, and (iii) 10-min irradiated sites.

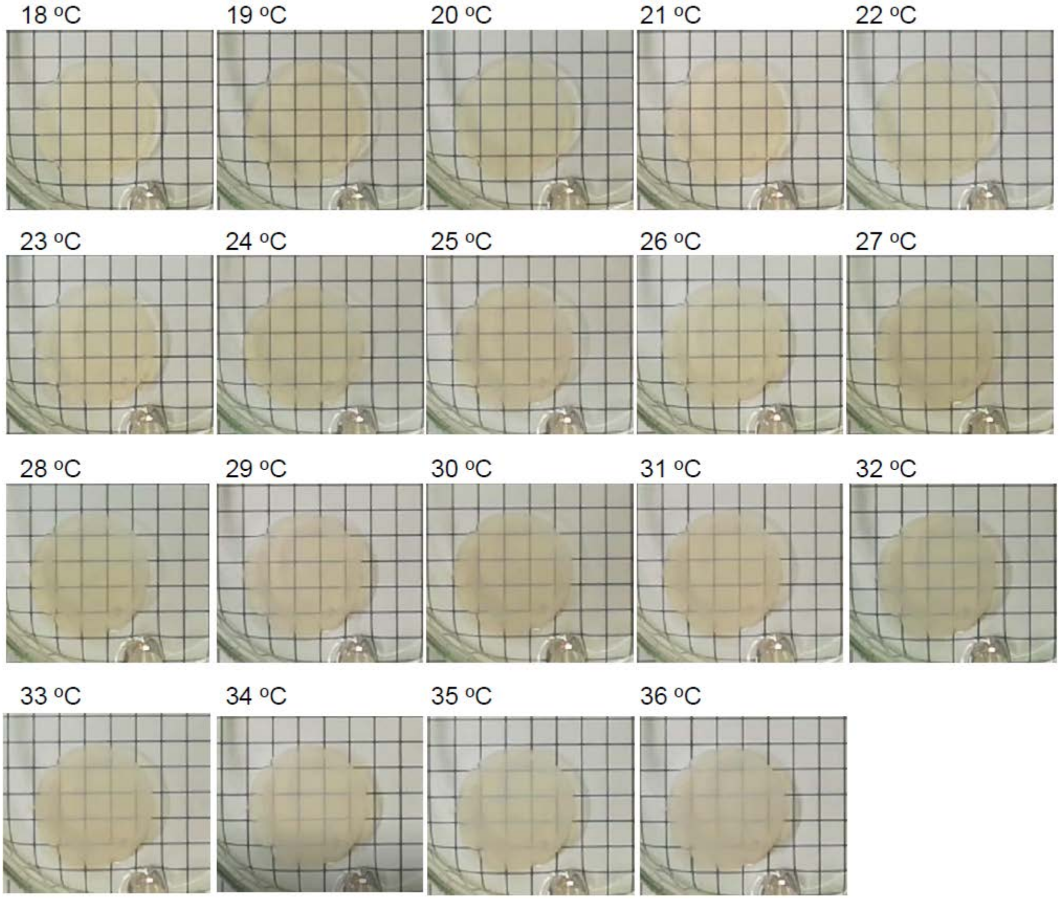

Figure S5. Top-view photographs of **P1a gel** with a pellet shape in chloroform during the heating process (ca. 3 °C/min). The values inserted above each picture denote the temperature Each square of the grid corresponds to 5 mm.

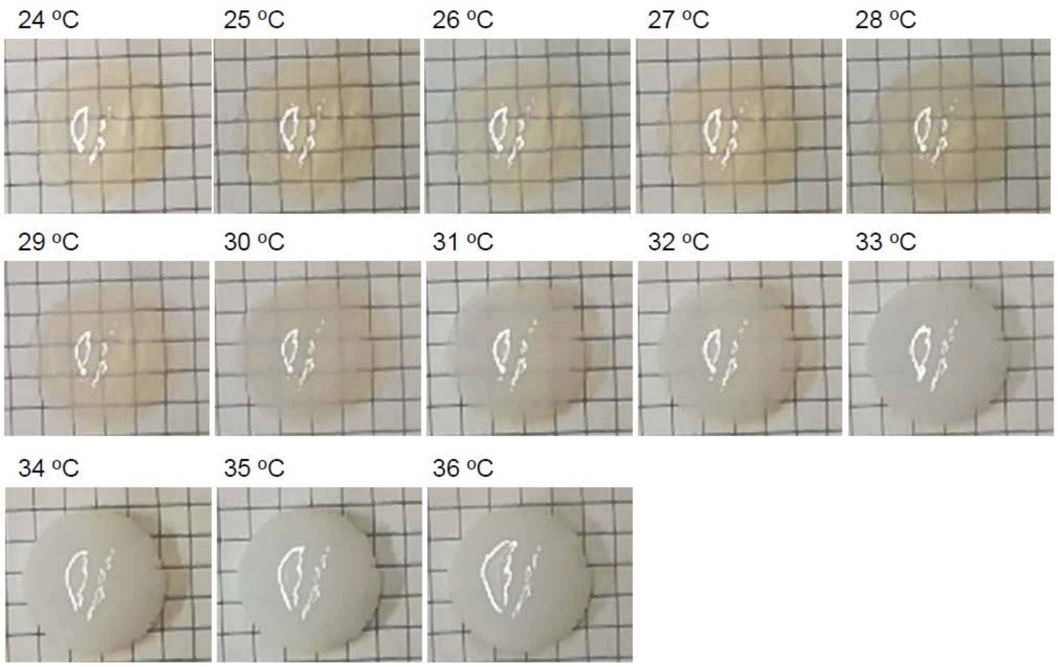

Figure S6. Top-view photographs of **P1b gel** with a pellet shape in chloroform during the heating process (ca. 3 °C/min). The values inserted above each picture denote the temperature Each square of the grid corresponds to 5 mm.

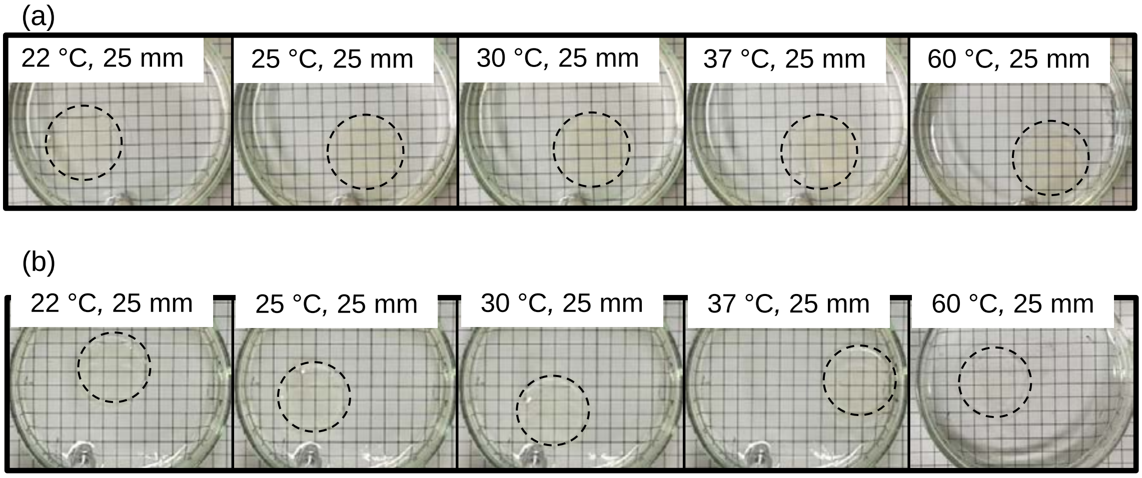

Figure S7. Top view photographs of (a) **P2a gel**, and (b) **P2b gel** with a pellet shape in chloroform during the heating process (ca. 3 °C/min). The values inserted in each picture denote temperature and the diameter of the gels. Dashed circles were drawn as visual guides for the outer shape of gels. Each square of the grid corresponds to 5 mm.

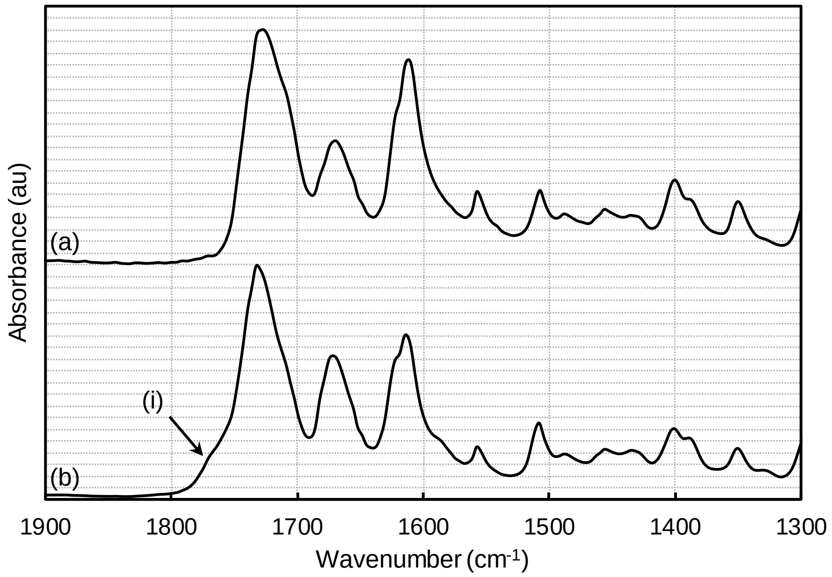

Figure S8. IR spectra of the surface of **P1b-gel** before (a) and after (b) photoirradiation. The arrow (i) shows a new shoulder peak at 1768 cm^{-1} assignable to a nonconjugated C=O group in the cyclobutene ring in the coumarin dimer.

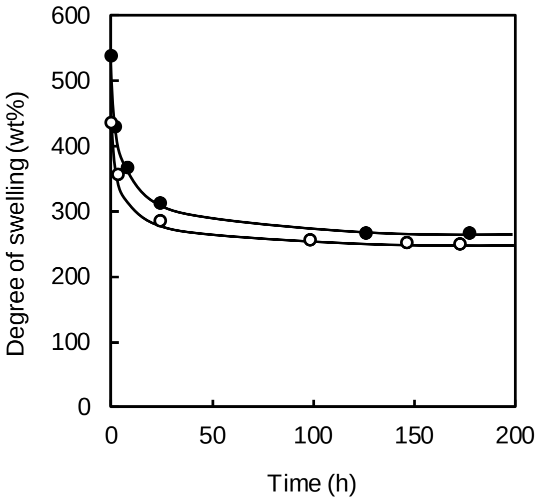

Figure S9. Time dependence of the degree of swelling of **P1b-gel** at 42 °C before (●) and after 5-min UV irradiation (○).

Table S1. Turbidity characteristics of **P1b gel** at each temperature.

Irradiation time (min)	Characteristics		
	-30 °C	22 °C	40 °C
0	Transparent	Transparent	Turbid
5	Transparent and Turbid	Turbid	Turbid
10	Turbid	Turbid	Turbid
